# Redox Imbalance and NOS‐Dependent Modulation of Superoxide in the Bladder Mucosa of Women With IC/BPS: A Preliminary Study

**DOI:** 10.1111/jcmm.71245

**Published:** 2026-06-11

**Authors:** Mariana G. de Oliveira, Edson Antunes, Gabriela Reolon Passos, Luiz Gustavo Oliveira Brito, Carlos Arturo Levi D'Ancona, Fabíola Z. Monica

**Affiliations:** ^1^ Laboratory of Pharmacology São Francisco University (USF) Sao Paulo Brazil; ^2^ Department of Pharmacology, Faculty of Medical Sciences University of Campinas Campinas Brazil; ^3^ Department of Obstetrics and Gynecology University of Campinas Faculty of Medical Sciences Campinas Brazil; ^4^ Department of Urology, Faculty of Medical Sciences University of Campinas São Paulo Brazil

**Keywords:** bladder pain syndrome, interstitial cystitis, nitric oxide synthase, phosphodiesterase, superoxide anion

## Abstract

Interstitial Cystitis/Bladder Pain Syndrome (IC/BPS) is a chronic condition characterized by bladder/pelvic pain and urinary symptoms, with poorly defined mechanisms and no reliable biomarkers. Oxidative stress, particularly reactive oxygen species (ROS), has been implicated in its pathophysiology, but direct human evidence remains scarce. Here, we evaluated ROS‐ and nitric oxide (NO)–related pathways in bladder mucosa from women with IC/BPS (*n* = 5) and controls (*n* = 7). Gene expression of NO–cGMP components, NADPH oxidases (NOX1–5), and antioxidant enzymes was assessed by qPCR. Baseline NO and superoxide ($O_{2}^{-}$) levels were quantified using histochemical assays, and the effects of pharmacological inhibitors of NOS, iNOS and NOX enzymes were examined. IC/BPS samples displayed increased expression of PDE5A (3.1‐fold) and p47phox (1.6‐fold), along with reduced SOD1 expression (0.39‐fold), consistent with an oxidative imbalance. Although baseline $O_{2}^{-}$ did not differ between groups, NOS inhibition with L‐NAME markedly reduced $O_{2}^{-}$ generation, with a greater effect in IC/BPS tissue (85% reduction). Conversely, selective iNOS inhibition (1400 W) increased $O_{2}^{-}$ levels in IC/BPS biopsies. These findings suggest altered NOS‐dependent redox regulation in the IC/BPS mucosa. Despite the limited sample size inherent to human biopsy studies, these results provide preliminary evidence of redox dysregulation in IC/BPS mucosa and highlight $O_{2}^{-}$ as a relevant mediator. They support further investigation into therapeutic strategies targeting NOS redox balance and cGMP signalling.

## 1 Introduction

Interstitial Cystitis/Bladder Pain Syndrome (IC/BPS) is a chronic, debilitating condition characterized by pelvic or bladder pain accompanied by urinary urgency, frequency and nocturia. Its pathophysiology remains poorly defined due to symptom heterogeneity, absence of reliable biomarkers and limited availability of well‐characterized human tissue [1]. Oxidative stress has been repeatedly implicated in IC/BPS, as reactive oxygen species (ROS) can impair bladder function through multiple pathways [1, 2, 3]. However, the contribution of specific ROS sources, particularly superoxide anion ($O_{2}^{-}$), remains unclear, and most human studies rely on indirect or nonspecific measures and lack pharmacological modulation.

Our previous work in a mouse model of IC/BPS demonstrated that excess $O_{2}^{-}$ is a key pathogenic driver and that disrupting NOS–redox balance contributes to disease features [4, 5]. Based on this, we hypothesized that redox and NO–cGMP pathways would also be altered in the bladder mucosa of women with IC/BPS. We therefore evaluated ROS‐related gene expression, basal redox mediators, and the effects of pharmacological inhibitors on $O_{2}^{-}$ generation. Here, we present preliminary human findings that parallel mechanisms identified in previous experimental models.

## 2 Materials and Methods

### 2.1 Samples

All procedures were approved by the University of Campinas Ethics Committee (88849718.9.0000.5404). Transurethral mucosal biopsies were obtained from women with IC/BPS (*n* = 5; 60.2 ± 6.2 years) confirmed by cystoscopy. Control samples were tumour‐free mucosa from women undergoing routine cystoscopy during sling procedures or intraoperative assessment, without clinical or cystoscopic signs of IC/BPS (*n* = 7; 51.3 ± 5.1 years). Tissues were collected into ice‐cold Krebs–Henseleit solution, processed within 1 h, segmented, snap‐frozen in liquid nitrogen, and stored at −80°C (either directly or in Tissue‐Tek OCT).

### 2.2 Real Time qPCR

Relative mRNA expression analysis was performed as previously described [3]. Qiagen QuantiTect primers were used for *Nos2* (QT00068740), *Nos3* (QT00043372), *Gucy1b3* (QT00013622), *Gucy1a3* (QT01001840), *Prkg* (QT00001638), *Pde5a* (QT00048167), *Nox1* (QT00025585), *Cybb* (QT00029533), *Nox4* (QT00057498), *Ncf1* (QT01004815), *Gapdh* (QT00079247) and *Rpl37* (QT00075012), the last two as housekeeping genes. Primers specific to *Sod1* (F:5′‐ACTGGTGGTCCATGAAAAAGC‐3′, R:5′‐AACGACTTCCAGCGTTTCCT‐3′) and *Nox5* (F:5′‐CGGTCTTTCGAGTGGTTTGTG‐3′, R:5′‐CCTCGGCCTGGTCCATCT‐3′) were obtained from Exxtend Technologies (Sao Paulo, Brazil). Relative expression was calculated using the 2^−ΔΔ*Ct*^ method.

### 2.3 Nitric Oxide Quantification (DAF‐2 Assay)

Transverse OCT‐embedded sections (12 μm) were incubated for 30 min in PBS containing 2 mM CaCl_2_ and 8 μM DAF‐2 (Thermo Scientific) at 37°C in the dark. Fluorescence images were acquired with a Nikon Eclipse Ti‐S microscope (10×, FITC filter) and analysed using ImageJ, normalized to area.

### 2.4 Superoxide Detection (DHE Assay)

OCT‐embedded sections (12 μm) were equilibrated in Hank's solution (10 min, 37°C) and pre‐incubated (30 min) with or without inhibitors of NOS (L‐NAME), iNOS (1400 W), xanthine oxidase (allopurinol), NOX1/4 (GKT137831), NOX2 (GSK2795039) or NOX5 (mellitin). Sections were then incubated with DHE (2 μM, 30 min). MnTMPyP (25 μM) served as a negative control. Images were acquired (Nikon Eclipse Ti‐S, 10×, rhodamine filter), analysed with ImageJ, normalized to area.

### 2.5 Statistical Analysis

Data are expressed as mean ± SEM. Normality was assessed by Shapiro–Wilk. Comparisons between groups used two‐tailed unpaired Student's *t*‐test, with *p* < 0.05 considered significant.

## 3 Results

### 3.1 Gene Expression Indicates Oxidative Imbalance in IC/BPS Bladder Mucosa

**FIGURE 1:**
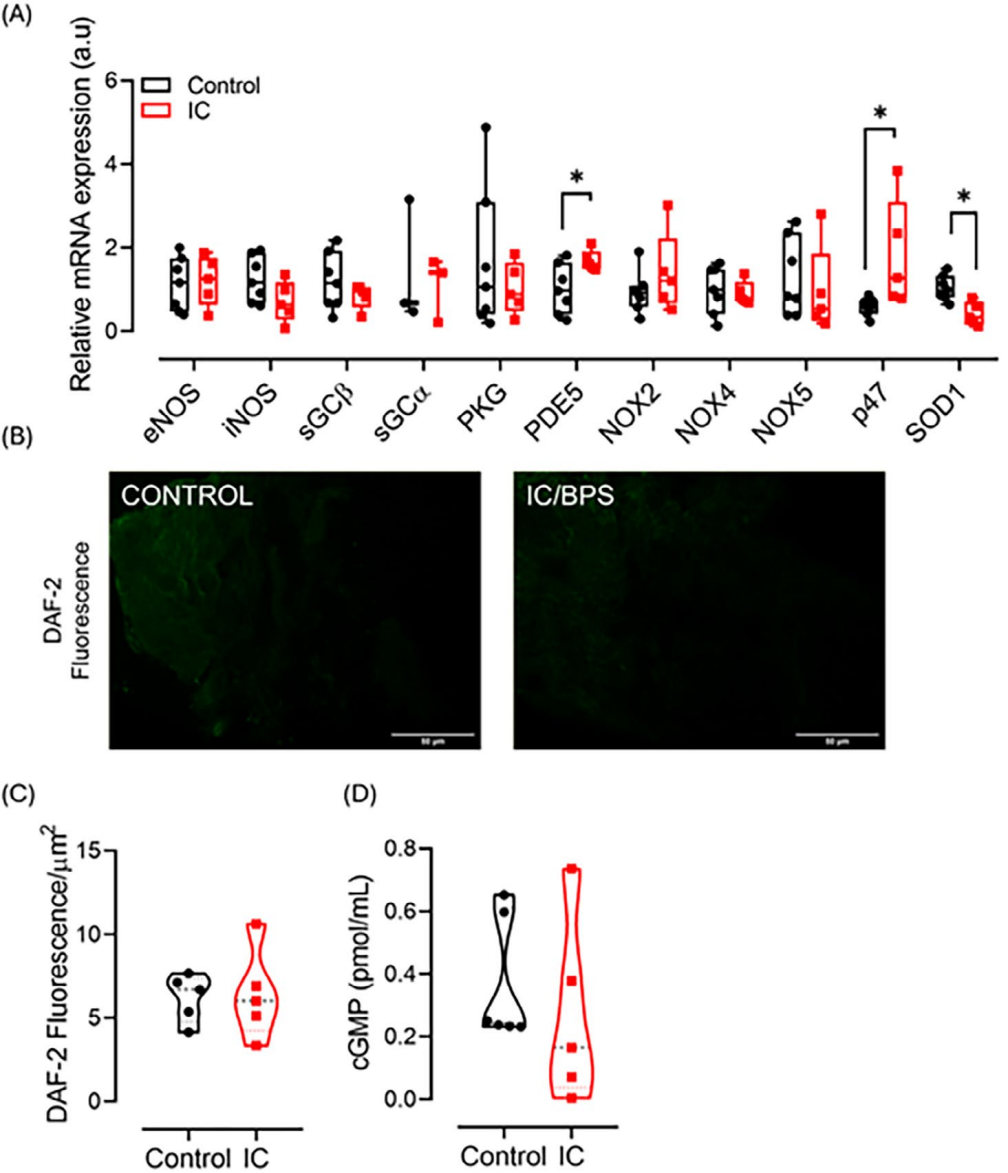
Relative mRNA expression levels in bladder mucosa samples from control and IC/BPS groups, measured by qPCR. *Nos2* (iNOS), *Nos3* (eNOS), *Gucy1b3* (sGC subunit β1), *Gucy1a3* (sGC subunit α1), *Prkg* (protein kinase cGMP dependent 1, PKG1), *Pde5a* (phosphodiesterase type 5A, PDE5A), NADPH oxidases (NOX) subunits NOX1 (*Nox1*), NOX2 (*Cybb*), NOX4 (*Nox4*) and NOX5 (*Nox5*), p47phox (*Ncf1*) and superoxide dismutase type 1 (*Sod1*). Data are presented as the mean ± SEM. *p < 0.05 in relation to control. Nitric oxide levels in bladder mucosa samples from control and IC/BPS groups, determined by DAF‐2 histochemistry assay, with representative images shown in (B) and fluorescence quantification in (C). Cyclic GMP levels are shown in (D). Data are presented as the mean ± SEM.

As shown in Figure 1A, gene expression analysis revealed a marked increase in PDE5 (approximately 3.1‐fold, *p* = 0.019) and p47phox (approximately 1.6‐fold, *p* = 0.015) in IC/BPS samples compared with controls. Conversely, SOD1 expression was reduced in IC/BPS samples, showing a decrease of about 0.39‐fold (*p* = 0.004, *n* = 5) relative to controls. No significant differences (*p* > 0.05) were observed in the mRNA expression levels of *eNOS, iNOS, sGCβ, sGCα, PKG, NOX1, NOX2, NOX4 or NOX5*.

### 3.2 Nitric Oxide Levels Are Not Altered in IC/BPS Bladder Mucosa

The baseline levels of NO, assessed using DAF‐2 fluorescence intensity (Figure 1B,C), did not differ significantly between bladder mucosa samples from control and IC/BPS groups (*p* > 0.05), indicating that NO production may not be altered in IC/BPS under non‐stimulated conditions. Accordingly, a non‐significant trend towards reduced cGMP levels (Figure 1D) in the IC/BPS group compared with controls (0.37 ± 0.08 vs. 0.27 ± 0.13 pmol/mg protein; *p* = 0.53). Among individual samples, three IC/BPS biopsies showed lower cGMP levels than the mean of the control group, while two were within the control range suggesting an overall trend towards reduction.

### 3.3 NOS Activity Modulates Superoxide Levels in IC/BPS Bladder Mucosa

**FIGURE 2:**
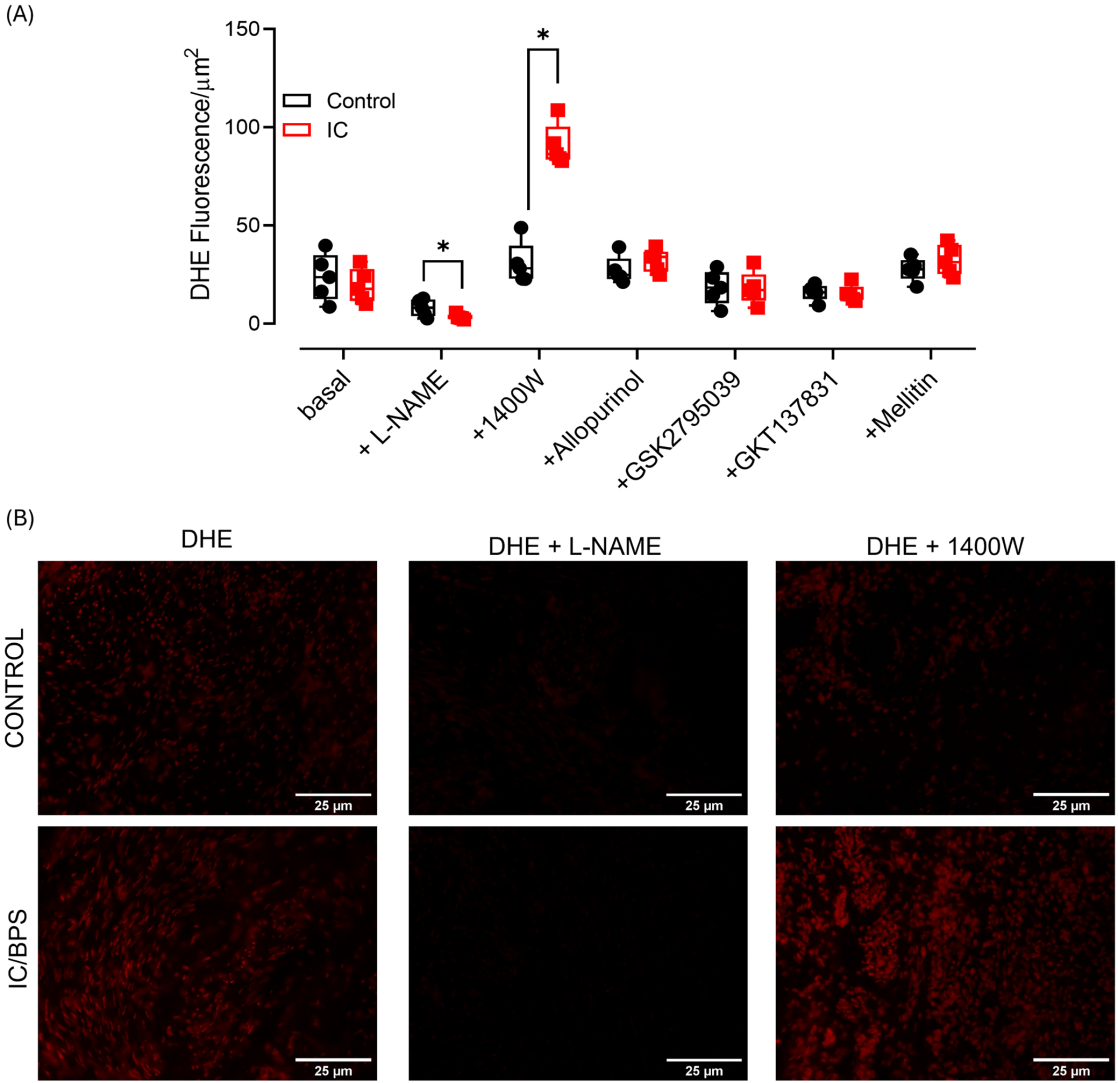
Superoxide levels (A), determined by DHE assay, in samples untreated or pre‐treated (30 min) with non‐selective or iNOS‐selective inhibitors of nitric oxide synthase (NOS) (L‐NAME or 1400 W, respectively), xanthine oxidase (allopurinol), NOX2 (GSK2795039), NOX1/4 (GKT137831) and NOX5 (mellitin). Representative DHE images (B) of samples untreated or pre‐treated (30 min) with L‐NAME or 1400 W. Data are presented as the mean ± SEM. *p < 0.05 in relation to control.

Figure 2A,B shows $O_{2}^{-}$ levels in samples from control and IC/BPS bladders. The baseline $O_{2}^{-}$ levels showed no significant difference (*p* > 0.05) between the control and IC/BPS samples. However, pre‐incubation with L‐NAME (30 min) significantly reduced $O_{2}^{-}$ generation in both groups: approximately 66% (*p* = 0.012) in control and about 85% (*p* = 0.001) in IC/BPS samples, compared to their respective baseline. Furthermore, the magnitude of the reduction induced by L‐NAME in IC/BPS samples was significantly greater than that in the control group (*p* = 0.009). Interestingly, pre‐incubation with the selective iNOS inhibitor 1400 W (30 min) remarkably increased $O_{2}^{-}$ generation in all samples from patients with IC by more than 3‐fold (*p* = 0.03). No significant changes (*p* > 0.05) in $O_{2}^{-}$ levels were observed in tissues treated with the selective NOX inhibitors GKT137831, GSK2795039 and mellitin, nor with xanthine oxidase inhibitor allopurinol.

## 4 Discussion

Our findings indicate that the bladder mucosa of women with IC/BPS exhibits a transcriptional and functional profile consistent with redox imbalance. The up‐regulation of p47phox, a regulatory cytosolic subunit involved in NOX activation, together with reduced SOD1 expression favours an environment in which $O_{2}^{-}$ is more likely to accumulate. Although NO levels assessed by DAF‐2 and cGMP showed only non‐significant trends towards reduction, even modest decreases in NO‐cGMP signalling may have physiological consequences, given the essential role of cGMP in urothelial sensory function [6]. ROS‐induced PDE5 upregulation has been described in cardiac and vascular tissues, where $O_{2}^{-}$ suppresses cGMP availability [7, 8, 9]. In our samples, the combination of elevated PDE5A expression and oxidative stress markers suggests that redox‐mediated modulation of the NO–cGMP pathway may occur in IC/BPS, although direct evidence is still needed.

A key observation was that L‐NAME markedly reduced $O_{2}^{-}$ levels, with a greater effect in IC/BPS. This finding is compatible with the idea of NOS‐derived $O_{2}^{-}$ formation, although not sufficient to prove NOS uncoupling, as direct markers of coupling status, such as BH_4_/BH_2_ ratio or NOS dimerization were not assessed here. In addition, because L‐NAME inhibits all NOS isoforms, a contribution of uncoupled eNOS from endothelial cells present in bladder mucosal vessels cannot be excluded [10]. Conversely, selective iNOS inhibition with 1400 W markedly increased $O_{2}^{-}$ generation in IC/BPS biopsies. One possibility is that reduced NO shifts the redox balance towards higher detectable $O_{2}^{-}$ or disrupts NOS isoform interactions, suggesting that iNOS inhibition may exert context‐dependent pro‐oxidant effects. Importantly, our previous mouse study [4, 5] did not show NOS‐derived $O_{2}^{-}$, highlighting species‐ or disease‐stage‐specific differences, underscoring the value of human samples.

This study is limited by its sample size and age differences between groups, constraints inherent to biopsy‐based IC/BPS research [11, 12, 13]. Nevertheless, consistent changes across assays strengthen confidence in the findings. Additional analyses, including protein expression, nitrotyrosine quantification, and NOS recoupling will be essential to refine mechanistic interpretations.

Overall, our findings highlight $O_{2}^{-}$ as a central redox mediator in IC/BPS mucosa, extending to human tissue the mechanisms we previously identified in our experimental IC/BPS model [3]. These preliminary human findings support the concept that redox dysregulation contributes to IC/BPS pathophysiology and suggest that interventions targeting $O_{2}^{-}$ production or restoring NOS redox balance or modulating downstream cGMP signalling.

## Author Contributions

**Mariana G. de Oliveira:** data curation, formal analysis, project administration, writing – review and editing, writing – original draft, visualization, methodology, investigation, conceptualization. **Edson Antunes:** conceptualization, funding acquisition, writing – original draft, writing – review and editing, methodology, formal analysis, project administration, supervision, resources, validation. **Gabriela Reolon Passos:** investigation, methodology. **Fabíola Z. Monica:** conceptualization, methodology, formal analysis, supervision, project administration, resources, funding acquisition, writing – original draft, writing – review and editing, investigation, visualization, validation. **Luiz Gustavo Oliveira Brito:** writing – original draft, investigation, methodology. **Carlos Arturo Levi D'Ancona:** investigation, methodology, writing – original draft.

## Funding

This work was supported by Fundação de Amparo à Pesquisa do Estado de São Paulo, 17/15175‐1, 18/09765‐3, 18/05956‐9 and 24/03517‐9.

## Conflicts of Interest

The authors declare no conflicts of interest.

## Data Availability

The data that support the findings of this study are available from the corresponding author upon reasonable request.
